# Animal models of critical illness in the Asia–Pacific region: current practices, shared challenges, and future directions

**DOI:** 10.1186/s40635-026-00874-9

**Published:** 2026-03-04

**Authors:** Yoshihisa Fujinami, Shuangqing Liu, Gianluigi Li Bassi, Marcin Osuchowski, Yongming Yao, John Fraser, Shigeaki Inoue

**Affiliations:** 1 Department of Emergency Medicine, Kakogawa Central City Hospital, Hyogo, Japan; 2 Department of Emergency and Critical Care Medicine, Wakayama Medical University, Wakayama, Japan; 3 Medical Innovation Research Division and Fourth Medical Center of the Chinese PLA General Hospital, Beijing, China; 4 Critical Care Research Group, The Prince Charles Hospital, Chermside, QLD Australia; 5 Institute for Molecular Bioscience, The University of Queensland, Brisbane, Australia; 6 Ludwig Boltzmann Institute for Traumatology, The Research Center in Cooperation With AUVA, Vienna, Austria

## Abstract

Animal models of critical illness span diverse species and experimental approaches, reflecting the biological complexity of severe disease states while being constrained by animal welfare requirements and country-specific regulatory, infrastructural, and workforce factors. Persistent challenges remain, including limited reproducibility, fragmented standards, and the need for ethical alignment across borders. This review examines these shared structural challenges in critical illness animal research across the Asia–Pacific region. While alternative and complementary methodologies are increasingly incorporated into preclinical research, their adoption remains uneven. We argue that alignment with globally recognized preclinical frameworks, including the 3Rs and disease-specific standards, such as MQTiPSS, is essential. This review discusses actionable strategies—centered on harmonized standards, shared resources, and international collaboration—to strengthen research rigor, support early career researchers, and enhance the translational relevance of critical illness animal research.

## Introduction

Critical illness encompasses life-threatening conditions, such as sepsis, trauma, burn, acute respiratory distress syndrome (ARDS), and post-cardiac arrest syndrome, all characterized by acute organ failure and multi-organ dysfunction. These syndromes are commonly encountered in intensive care units worldwide and remain associated with high mortality and poor outcomes [1–3]. Contemporary intensive care has improved survival through advances in organ support and infection control; however, the pathophysiology of critical illness remains incompletely understood, as immune, metabolic, and inflammatory responses evolve dynamically across multiple biological levels [4, 5]. Many key mechanistic insights require invasive and time-resolved sampling that is not feasible in human studies. In this context, animal-based experimental research plays a pivotal role in elucidating disease mechanisms and informing therapeutic development [6–8]. Well-characterized animal models remain a cornerstone of mechanistic discovery and preclinical validation, and, therefore, must adhere to the highest standards of scientific rigor and ethical responsibility. Nevertheless, due to country-specific differences in ethical frameworks, resource availability, and institutional infrastructures, these ideals are not uniformly achieved.

This journal has previously highlighted that although animal research of critical illness remains essential to biomedical advancement, it inherently entails structural and ethical challenges that warrant deliberate recognition and systematic resolution [8]. Building on prior discussions in this journal, this review examines structural, ethical, and translational challenges in animal models of critical illness across the Asia–Pacific region, with a focus on Australia, China, and Japan, and proposes directions for improved rigor and international collaboration.

### Current status: infrastructure and modeling directions

In the Asia–Pacific region, rodent-based models remain central to preclinical research on critical illness [9], with different experimental paradigms—including cecal ligation and puncture (CLP), lipopolysaccharide (LPS)-based endotoxemia, and experimental lung injury models—used selectively to decipher distinct aspects of sepsis and acute respiratory distress syndrome (ARDS) pathophysiology [10–16]. Their widespread use reflects cost-effectiveness, genetic tractability, and the availability of standardized experimental protocols. In contrast, medium-sized animal models such as pigs, dogs, and sheep provide greater physiological similarity to humans, particularly in cardiovascular and respiratory systems, and, therefore, offer higher translational potential. Large-animal models have played a critical role in advancing physiologically grounded ventilation strategies and device development, contributing to the translation of lung-protective approaches into clinical practice [17–21]. Porcine models are also particularly valuable for studying ARDS, extracorporeal membrane oxygenation (ECMO), trauma, and device-related interventions because of their comparable organ size and immune responses [22, 23]. Nevertheless, the use of large species models remains limited due to high maintenance costs, the need for specialized infrastructure and personnel, and increasing ethical and regulatory constraints associated with larger vertebrate species [24].

Across the Asia–Pacific region, marked disparities persist in infrastructure, model innovation, and regulatory oversight related to animal model research (Table 1). These disparities are further highlighted by the emergence of alternative approaches, which, despite their promise, remain limited by incomplete representation of systemic physiology, challenges in standardization, and high implementation costs. While Australia, North America, and the European Union have advanced coordinated initiatives to promote non-animal methodologies, many Asian countries—including China and Japan—lack centralized strategies or sustained public investment, risking further widening global inequities in research capacity. Against this backdrop, strategic investment in infrastructure, combined with academic–industry partnerships and international collaboration, may enable the complementary use of medium-sized animal models and strengthen the translational relevance of critical illness research.

**Table 1:** Animal experimentation management systems in Australia, China, and Japan

	Australia	China	Japan
History	The *Australian code of practice for the care and use of animals for scientific purposes* (the Code) by NHMRC since 1969	A specialized academic organization founded in the 1980s; since then, national conferences convened, ministerial regulations and a state-level statute promulgated	A specialized agency established in 1952; institutional development progressed from the 1970s onward
Regulatory agencies	NHMRC, nation-wide	State Council (national statutes), MOST (centralized oversight), SAC (national standards), CALAS (academic coordination)	CIEA (Public Interest Corporation) JALAS (Academic Society)
Type of oversight	Mandatory	Central–local coordinated regulation, statutory/regulatory supervision, specialized agency oversight, and academic-supported oversight	Voluntary
Legal regulations	The Australian Code is legally compulsory across Australia, with each state and territory adopting it with local variations. Non‑compliance can result in monetary penalties and/or imprisonment	Non-legally binding administrative regulations and national standards; integrating mandatory licensing, voluntary institutional implementation	Defragmented, non-legally binding; combination of national guidelines and voluntary institutional practices

*NHMRC* National Health and Medical Research Council, *MOST* Ministry of Science and Technology, *SAC* Standardization Administration of China, *CALAS* Chinese Association for Laboratory Animal Sciences, *CIEA* Central Institute for Experimental Animals, *JALAS* Japanese Association for Laboratory Animal Science

### Country-specific characteristics and challenges

In all three countries, the principles of the 3Rs (replacement, refinement, and reduction) constitute a foundational framework for animal experimentation. Australia represents a highly standardized and legally enforced model in which compliance with the 3Rs is mandated through state and territory legislation. China has developed a centrally coordinated regulatory system in which the 3Rs are embedded within administrative statutes, although implementation may vary by institution and region. In contrast, Japan relies on a guideline-based framework that emphasizes institutional responsibility and ethical self-regulation rather than legal compulsion.

**Fig. 1:**
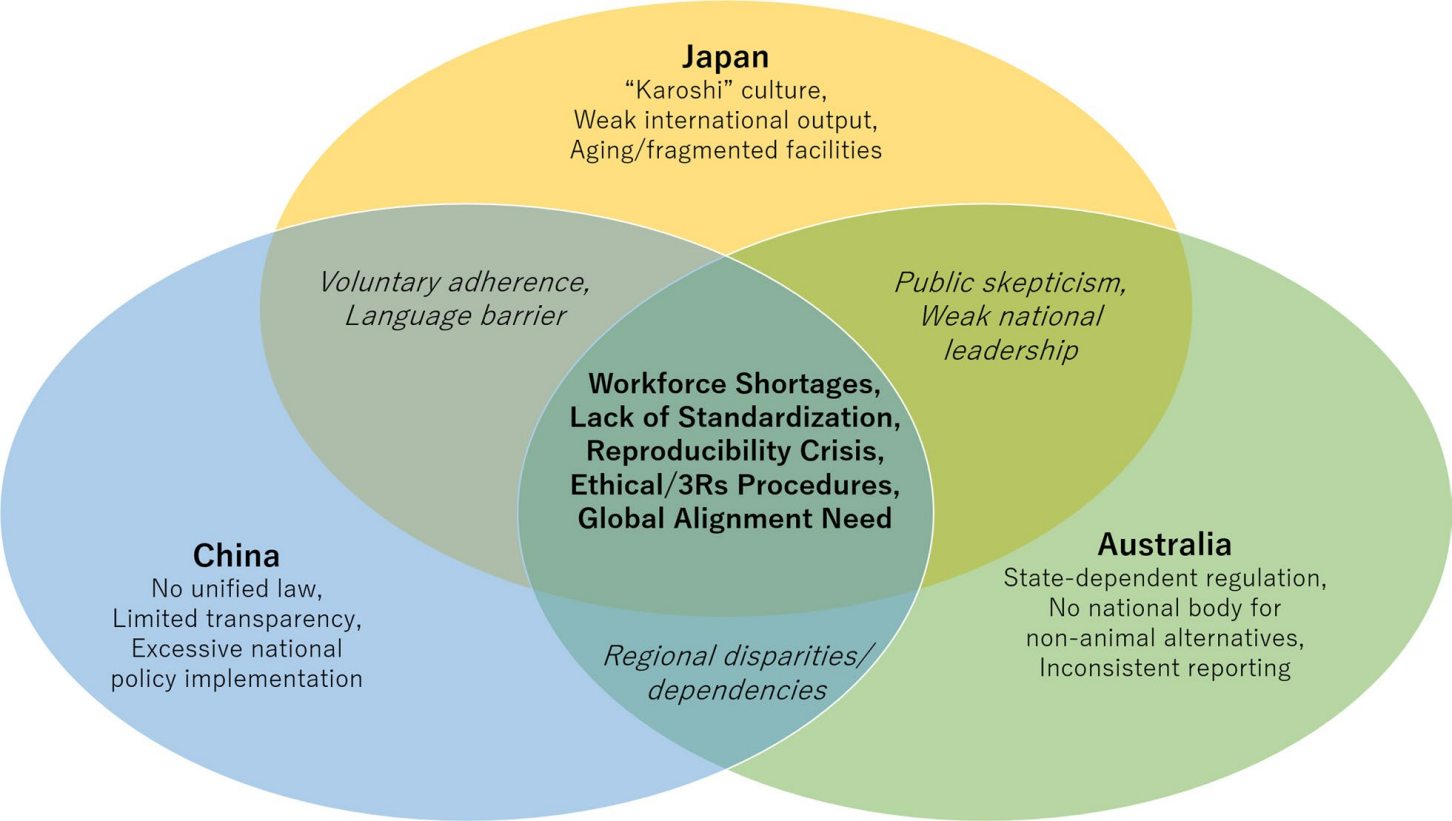
Country-specific and shared challenges in animal model research across Australia, China, and Japan. The Venn diagram summarizes regulatory, structural, and societal challenges affecting animal experimentation in the three countries discussed in Chapter 3. The overlapping center identifies the joint shortcomings/challenges signifying the need for coordinated international action to at least partly remedy them

Key differences in animal experimentation systems and the 3Rs compliance across the three countries are summarized in Table 2. Beyond regulatory frameworks, animal-based research in critical care medicine is also shaped by institutional priorities, infrastructure, workforce capacity, and societal expectations, as illustrated in Fig. 1.

**Table 2:** Differences in animal experimentation and the 3Rs compliance across three countries

Topic	Australia	China	Japan
Definition of laboratory animals	All live non-human vertebrates + cephalopods	Artificially-bred, microbiologically controlled animals	Live vertebrate animals
Legal status of the 3Rs	Explicitly embedded in legally binding state/territory laws	Mandatory under national statutes and administrative regulations	Promoted through national guidelines; limited legal enforceability
Enforcement	Strong legal enforcement via state legislation	Strong administrative enforcement with penalties	Institutional self-regulation
Justification for lack of alternatives	Clearly required and formally documented	Explicitly required, but variable rigor	Required, but depth varies by institution
Special regulation of primates	Mandatory ethical review; highly restrictive	Licensed, restricted use	Strict ethical scrutiny
Regulation of GM animals	Regulated under gene technology legislation	Regulated under biosafety frameworks	Regulated under the Cartagena Act

#### Australia

In Australia, all animal scientific use requires prior approval by an institutional animal ethics committee operating under the legally enforced Australian Code of Practice for the Care and Use of Animals for Scientific Purposes [25]. Although the Code is nationally recognized, definitions and reporting requirements vary across states—for example, some jurisdictions exclude fish from statutory protection—complicating the collection of consistent national data on animal use. This heterogeneity in reporting practices limits accurate national benchmarking and may hinder evidence-based decisions, including public investment in nonanimal research alternatives.

In parallel, structured career pathways for clinician–scientists in intensive care medicine remain limited, and heavy clinical workloads discourage sustained engagement in laboratory-based research. Large-animal experiments require continuous supervision by highly skilled teams, often involving six to eight investigators for prolonged protocols, placing substantial demands on human resources. Consequently, many institutions rely on international clinician–scientists to sustain large-animal research programs, although long-term retention of these specialists remains challenging.

Funding success rates for early stage laboratory research have also declined steadily in recent years, a trend accentuated since the COVID-19 pandemic. The success rate of the Australian Research Council’s Discovery Projects fell from 20.0% in 2021 to 12.9% in 2025 when including expressions of interest, while NHMRC Ideas Grants declined from approximately 11% in 2022 to around 8% in 2025. As a result, securing supplementary funding from non-profit foundations and industry partners has become increasingly necessary, particularly for resource-intensive large-animal studies with prolonged follow-up.

#### China

Animal experimentation in China is governed by a multi-tiered system of administrative statutes and sector-specific regulations coordinated across multiple ministries, rather than by a single comprehensive national law [26, 27]. Ethical and operational standards are further refined through national guidelines that embed the 3Rs principle and require institutional animal ethics committees; however, these frameworks remain largely administrative and lack the legal enforceability and uniformity seen in EU or US regulations [28–30]. As a result, the structure, independence, and transparency of IACUCs vary widely across institutions, with limited requirements for public disclosure of protocols or welfare outcomes, undermining accountability and consistency.

A major structural challenge is the shortage of specialized personnel, with only approximately 1% of the laboratory animal workforce formally trained in laboratory animal science, and limited systematic education in animal welfare and ethics [27, 31]. Heavy clinical workloads discourage clinician engagement in basic research, while underqualified animal care staffing, aging infrastructure, and uneven access to advanced monitoring and multi-omics platforms collectively undermine experimental rigor, welfare standards, and reproducibility. These challenges are compounded by a publication-oriented evaluation system that prioritizes molecular novelty over clinical relevance and translational value.

Increasing global integration has intensified pressure to harmonize Chinese practices with international standards. China’s export of research non-human primates—previously accounting for over 70% of global supply—has declined by approximately 96%, exposing vulnerabilities related to animal welfare, logistics, and biosecurity [32]. In parallel, international regulatory shifts, including bans on animal testing in certain markets, have accelerated the need to develop non-animal alternatives; however, financial support and mechanisms for shared data platforms and resource co-development remain insufficient [30].

#### Japan

The most systemic challenge in Japan’s animal model research lies in its regulatory and institutional framework. Oversight relies on a combination of national guidelines and voluntary institutional practices rather than legally binding mandates. A major step toward systematization was the issuance of the Basic Guidelines for the Conduct of Animal Experiments in 2006 [33], which emphasize ethical oversight and institutional responsibility. These guidelines promote the principles of the 3Rs and recommend the establishment of IACUC-equivalent review bodies; however, unlike the legally mandated frameworks in the US and European Union, such structures are not legally required in Japan [34]. Nevertheless, the broad influence of these guidelines has led nearly all medical schools, pharmaceutical companies, and approximately one-third of laboratory animal breeders to establish independent animal experimentation committees [35].

Beyond governmental guidance, the Central Institute for Experimental Animals (CIEA) and the Japanese Association for Laboratory Animal Science (JALAS) play central roles in promoting standardized infrastructure, ethical review, and alignment with international accreditation frameworks, such as AAALAC International. Despite this structured yet largely voluntary system, the absence of legally enforceable requirements results in institutional variability in review rigor and transparency, posing challenges for international collaboration that increasingly demands formal IACUC-equivalent documentation.

A second major structural challenge is the shortage of human resources in animal model research. The lack of institutional recognition of basic research as part of healthcare professionals’ formal duties forces many clinicians to conduct research during personal time. This burden is amplified by the demanding clinical environment in Japan, where excessive workloads—often referred to as “karoshi” (death from overwork)—limit sustained research engagement [36]. Consequently, clinicians seeking long-term involvement in basic research often pursue opportunities abroad, raising concerns about talent outflow.

Additional barriers include an aging research workforce, declining interest in basic medical research among younger scientists, heavy reliance on competitive public funding, and limited international visibility. Collectively, these challenges underscore the need for long-term, coordinated strategies to develop human resources and maintain Japan’s competitiveness in animal model research.

### Research focus areas, translational successes and emerging strategies

The scientific value of animal experimentation extends beyond direct pathophysiological insights or immediate therapeutic development. These contributions include improving reproducibility, developing experimental platforms, and generating conceptual frameworks that may not yield immediate clinical applications. Although thematic overlaps exist, each country has developed distinct research focus areas supported by specific preclinical models. Table 3 summarizes representative national preclinical studies that have contributed to advances across multiple domains of critical illness research.

**Table 3:** Representative experimental models of critical illness in Australia, China, and Japan

Country	Model type	Focus area
Australia	ARDS/ALI (by smoke inhalation, oleic acid, endotoxin, pancreatitis, transfusion, fluid overload, SARS-CoV-2) Rat/sheep	Model development [39, 51, 53, 54, 57] Pathophysiology and phenotyping [47, 50, 95] Therapeutic interventions [55, 56]
	Burn Sheep/pig	Wound healing [37, 38]
	Hemorrhagic and/or traumatic shock (volume, MAP-controlled) Sheep	Coagulopathy [40] Organ damage [41]
	Septic shock (Endotoxin, *E.coli* bacteremia) Sheep	Model development [42, 96] Acute kidney injury [58, 63–65] Resuscitation strategies [43, 59–61] Therapeutic interventions [66–69]
	Heart transplantation Sheep	Brain-stem death donation/Transplant model development [44] Heart preservation [45]
	ECMO (veno-arterial, veno-venous) Sheep	Model development [48, 49] Therapeutic interventions [97, 98]
	Cardiopulmonary bypass Sheep	Acute kidney injury: pathophysiology and treatment [63, 64, 71, 72, 77–80] Neuro-inflammation: pathophysiology and treatment [99–101]
China	Abdominal sepsis (CLP, FS, "two-hit" model, LPS endotoxemia) Mouse/pig/dog	Therapeutic interventions [102–105] Hemodynamic monitoring [106] Immune modulation mechanisms [107–111] Cell/organelle damage [112–115] Organ dysfunction [116–119] Immune subtyping [120, 121]
	Hemorrhagic and/or traumatic shock (volume, MAP-controlled) Rat/dog/pig	Pathophysiological changes/mechanism [122–126] Therapeutic interventions [127, 128]
	ARDS/ALI (i.t./i.n. LPS, CLP-induced, repeated lung/BAL) Rat/dog/pig/sheep	Heterogeneity of immune cells [129, 130] Therapeutic interventions [131–134] Immune regulatory mechanisms [135–137]
	AKI (sepsis, rhabdomyolysis, crush syndrome, ischemia–reperfusion injury) Rat/pig/rabbit	Cell injury/death mechanism [138–140] Heterogeneity of immune cells [141] Therapeutic interventions [142–144]
	Cardiac arrest Pig/mouse	Therapeutic interventions [145–147] Organ protection [148–150] Novel cardiopulmonary resuscitation strategy [151]
Japan	Abdominal sepsis (CLP, FS) Mouse	Immune regulatory mechanisms [152–154] Organ protection/therapeutic interventions [155–158] Post-Intensive Care Syndrome (PICS) [159–163]
	Hemorrhagic shock (Volume, MAP-controlled) Pig/rat	Immune function decline following massive transfusion [164] Pharmacokinetics and pharmacodynamics [165] Organ protection/therapeutic interventions [166, 167]
	Ischemia–reperfusion injury Pig/rat	Organ protection/therapeutic interventions [168–170] Focal cerebral ischemia (Methodology) [171]
	ARDS/VILI (CLP, Airway pressure load, Fluid load) Pig/rat	Model development [172] Pathophysiology/therapeutic interventions [173–175]
	Subarachnoid haemorrhage Mouse/rat	Model development/refinement [176] Neuroprotection [177] Neuro-cardiac signaling [178]
	Trauma (Blast injury) Mouse/rat	Brain injury [179, 180] Lung injury [181, 182] Inner ear injury [183, 184]

*ARDS* acute respiratory distress syndrome, *ALI* acute lung injury, *SARS-CoV-2* severe acute respiratory syndrome coronavirus 2, *MAP* mean arterial pressure, *ECMO* extracorporeal membrane oxygenation, *CLP* cecal ligation and puncture, *FS* fecal slurry, *i.t.* intratracheal, *i.n.* intranasal, *BAL* bronchoalveolar lavage, *VILI* ventilator-induced lung injury, and *SOD* superoxide dismutase

#### Australia

**Fig. 2:**
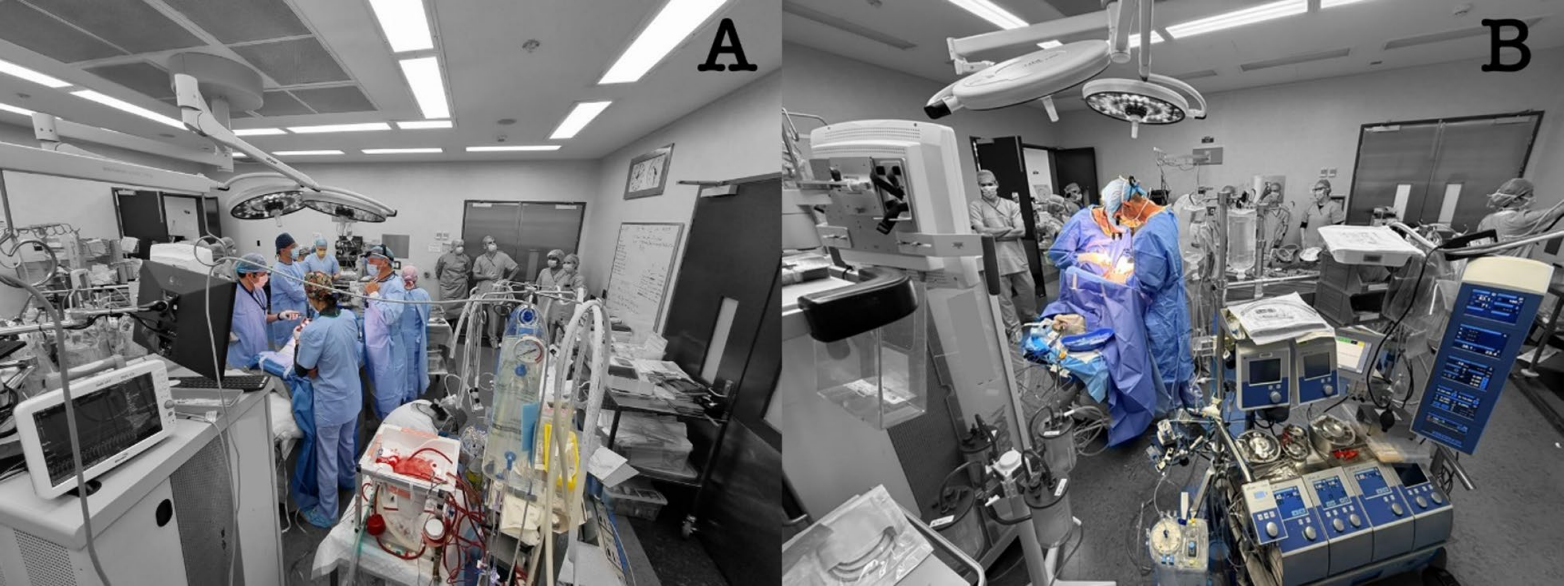
Preclinical Innovative Medical Engineering Laboratory (PRIMELab) in Chermside, QLD, Australia. PRIMELab is part of the Critical Care Research Group, located within the Medical Engineering Research Facility at Queensland University of Technology on The Prince Charles Hospital campus. **A** A multidisciplinary team—comprising intensivists, cardiac surgeons, anesthetists, cardiologists, perfusionist, and scientists—is shown video-recording heart explant after 24 h from the development of brainstem death. The Hypothermic Oxygenated Perfusion (HOPE) prototype device for donor heart preservation is visible in the lower right of the image [43]. **B** After several hours of donor heart preservation, the heart is transplanted into a new animal (recipient) supported by cardiopulmonary bypass, as depicted in the image

Over the past two decades, Australia has developed strong expertise in critical illness modeling through internationally recognized centers of translational research. The Critical Care Research Group (CCRG), through its Preclinical Innovative Medical and Engineering Laboratory (PRIMELab) (Fig. 2), established sophisticated large-animal platforms capable of capturing complex, multi-organ critical illness. These models enabled detailed physiological and metabolic monitoring and supported translational advances across trauma, sepsis, lung injury, and transplantation research, several of which directly informed subsequent clinical trials [37–46]. The group also contributed substantially to ARDS and ECMO research through ovine models designed to replicate clinically relevant cardiopulmonary physiology [47–49].

At Flinders University, the Lung Lab developed a range of translational small-animal models of direct and indirect lung injury, generating mechanistic insights into inflammatory lung damage and fluid-induced exacerbation of pulmonary injury, with direct implications for ICU practice [50–57]. The Florey Institute’s Translational Cardiovascular and Renal Research Group established advanced non-anesthetized ovine models of gram-negative sepsis, enabling continuous multi-organ monitoring. Seminal findings demonstrated a dissociation between renal blood flow and acute kidney injury and identified angiotensin II as an effective vasopressor that improved renal function, contributing to the clinical development of angiotensin II (Giapreza) for vasodilatory shock [58–62]. Subsequent studies established urinary oxygen tension as a biomarker of renal medullary hypoxia and AKI and informed optimized perfusion strategies during cardiopulmonary bypass, later validated clinically [63–82].

#### China

Academic institutions, national research centers, and key laboratories serve as the primary drivers of animal-based basic medical research in China, supported by specialized technical teams and graduate training programs. Using gene-edited animal models, Chinese researchers have elucidated the roles of multiple regulated cell death pathways—including pyroptosis, ferroptosis, and autophagy—in immune dysregulation during sepsis [15, 83, 84]. In parallel, Chinese laboratories have both adopted internationally established models and developed context-specific animal models tailored to national research priorities and resource settings [85–87].

In xenotransplantation, Chinese researchers have developed pig-to-macaque heart and kidney transplantation models using gene-edited, cytomegalovirus-negative pigs, achieving over 1 year of graft survival and overcoming hyperacute rejection [88]. Notably, China also led the first successful transplantation of a multi-gene-edited pig liver into a brain-dead human, with sustained graft function for 10 days, marking a major milestone in clinical translation [89].

Leveraging non-human primate resources and CRISPR–Cas9 technology, Chinese researchers have established several world-first disease models, including a non-human primate model of ataxia–telangiectasia and a spontaneous self-injurious behavior macaque model, addressing previously inaccessible questions in neurodegenerative and psychiatric research [90, 91]. These efforts are supported by national primate facilities, biobanks, and coordinated training and funding programs [92].

Recognizing the heterogeneity of sepsis models and their limited clinical representativeness [93], China is increasingly adopting a translation-oriented strategy that emphasizes clinically relevant disease trajectories and supportive care. This strategy integrates artificial intelligence (AI) and big data analytics to link animal phenomics with clinical data sets, enabling the identification of molecular subtypes, prognostic biomarkers, and therapeutic targets and thereby improving translation from basic discoveries to clinical applications.

#### Japan

In Japan, animal experimentation in drug discovery is largely driven by pharmaceutical companies with substantial investment in infrastructure and genetically engineered models, whereas basic medical research is primarily led by university-based laboratories with strong traditions in microbiology, immunology, and neuroscience [34]. Within severe illness research, rodent-based models of sepsis, ARDS, ischemia–reperfusion injury, and hemorrhagic shock are widely used, with increasing efforts to enhance clinical relevance by incorporating aging, comorbidities, and multimodal supportive care. Importantly, many experimental studies are conducted by clinician–scientists, including early career trainees, allowing research questions to arise directly from bedside observations and enhancing translational alignment of model design (representative examples are summarized in Table 3). Japan has also begun to integrate engineering, imaging, and computational approaches into animal research. High-resolution in vivo imaging and multi-omics platforms are increasingly used to characterize temporal organ injury dynamics, while early applications of artificial intelligence to animal behavior analysis, phenotyping, and protocol optimization are emerging as national data infrastructure initiatives expand.

Nonetheless, structural challenges related to facilities, funding, and human resource development remain substantial. In response, academic organizations—including the Japanese Society of Intensive Care Medicine (JSICM) and the Japanese Society for Education and Promotion of Intensive Care in Clinical Medicine (JSEPTIC)—are promoting clinician engagement in foundational research through education programs, industry–academia collaboration, and integration of critical care data platforms with biomedical research. These initiatives increasingly incorporate data science and AI-driven approaches, although their application to animal model research remains at an early stage [94].

### Research reality and public perception

#### Australia

Australia ranks among the leading countries worldwide in the use of animal models for biomedical research [185]. However, reporting is conducted at the State and Territory level rather than through a unified national system, resulting in heterogeneous data and limited standardized benchmarking across institutions [186]. Although continuous efforts are being made to reduce and replace animal use in medical research, Australia lacks a dedicated national body for developing alternative methods. In this context, New South Wales recently launched the Non-Animal Technologies Network (NAT-Net), allocating $4.5 million to support competitive grants aimed at reducing and replacing animal use in medical research [187].

Public support for animal research in Australia is largely grounded in transparency and assurances that studies are conducted humanely, for legitimate medical purposes, and only when no viable alternatives exist. Nonetheless, increasing environmental awareness and recognition of animal sentience have intensified ethical tensions between animal welfare concerns and the continued instrumental use of animals in research. Animal welfare regulation is administered by state and territory governments under the NHMRC Code, which is currently under review, with oversight provided by the Animal Welfare Committee (AWC) [25]. The AWC includes scientific experts, animal welfare specialists, and community representatives to ensure ethical governance and societal accountability [188].

Despite these governance structures, a significant gap remains in understanding Australian public attitudes toward animal research. While previous studies have examined the perspectives of researchers, ethics committee members, and animal welfare professionals, comprehensive assessments of broader public opinion are lacking. A 2022 report commissioned by the Australian and New Zealand Council for the Care of Animals in Research and Teaching (ANZCCART) provided initial insights into public attitudes, highlighting opportunities to improve public engagement on regulatory processes, species selection, and the 3Rs—particularly replacement and reduction [189]. However, the survey did not clarify the depth or type of information the public seeks. Accordingly, a deeper understanding of the social and cultural factors shaping public attitudes is urgently needed to inform effective communication strategies and sustain societal trust in animal-based research.

#### China

Despite the challenges outlined above, Chinese researchers broadly recognize the necessity of animal research for advancing biomedicine, developing new therapies, and supporting national scientific and technological innovation. In parallel, laboratory animal ethics and welfare education have been increasingly promoted, emphasizing the 3Rs principles and reinforcing the view that standardized animal research enhances both ethical compliance and scientific rigor [190].

The Chinese public generally maintains a supportive attitude toward animal research, recognizing its role in scientific and technological progress and social development. However, advocacy by animal protection organizations has contributed to more critical views among certain groups, particularly regarding animal welfare and the perceived cruelty of experimentation [191]. While such perspectives have not directly impeded the overall progress of animal research, they have increased societal pressure on researchers and heightened expectations for transparency and public engagement. Traditional philosophical frameworks in China, including Confucianism, Taoism, and Buddhism, have long influenced perceptions of human–animal relationships, often emphasizing human moral responsibility rather than the intrinsic value of animals. Consequently, humane treatment of animals is frequently viewed as an expression of personal moral cultivation rather than recognition of animals’ inherent rights. As in other regions, sustained and targeted public science communication across diverse demographic groups is essential to improve understanding of the life-saving contributions of animal research and to transparently convey ongoing efforts to enforce animal welfare standards and the 3Rs.

#### Japan

Public perception of animal research in Japan is characterized by ambivalence: while more than half of respondents acknowledge its necessity for advancing medical technology and medical education, a majority simultaneously regard animal experimentation as a cruel practice [192]. Although a direct causal link with research productivity remains unclear, such negative perceptions may be sufficient to discourage early career researchers from engaging in animal-based studies. Surveys further indicate limited public awareness of regulatory safeguards, including the 3Rs principles and ethical review processes, contributing to a persistent gap between researchers’ efforts to ensure rigor and public understanding of how animal experiments are conducted in practice. As societal expectations for ethical justification continue to rise in Japan, this disconnect underscores the need for more proactive science communication and institutional transparency to sustain public trust and support the next generation of researchers.

### Toward a stronger footing: what is needed?

**Fig. 3:**
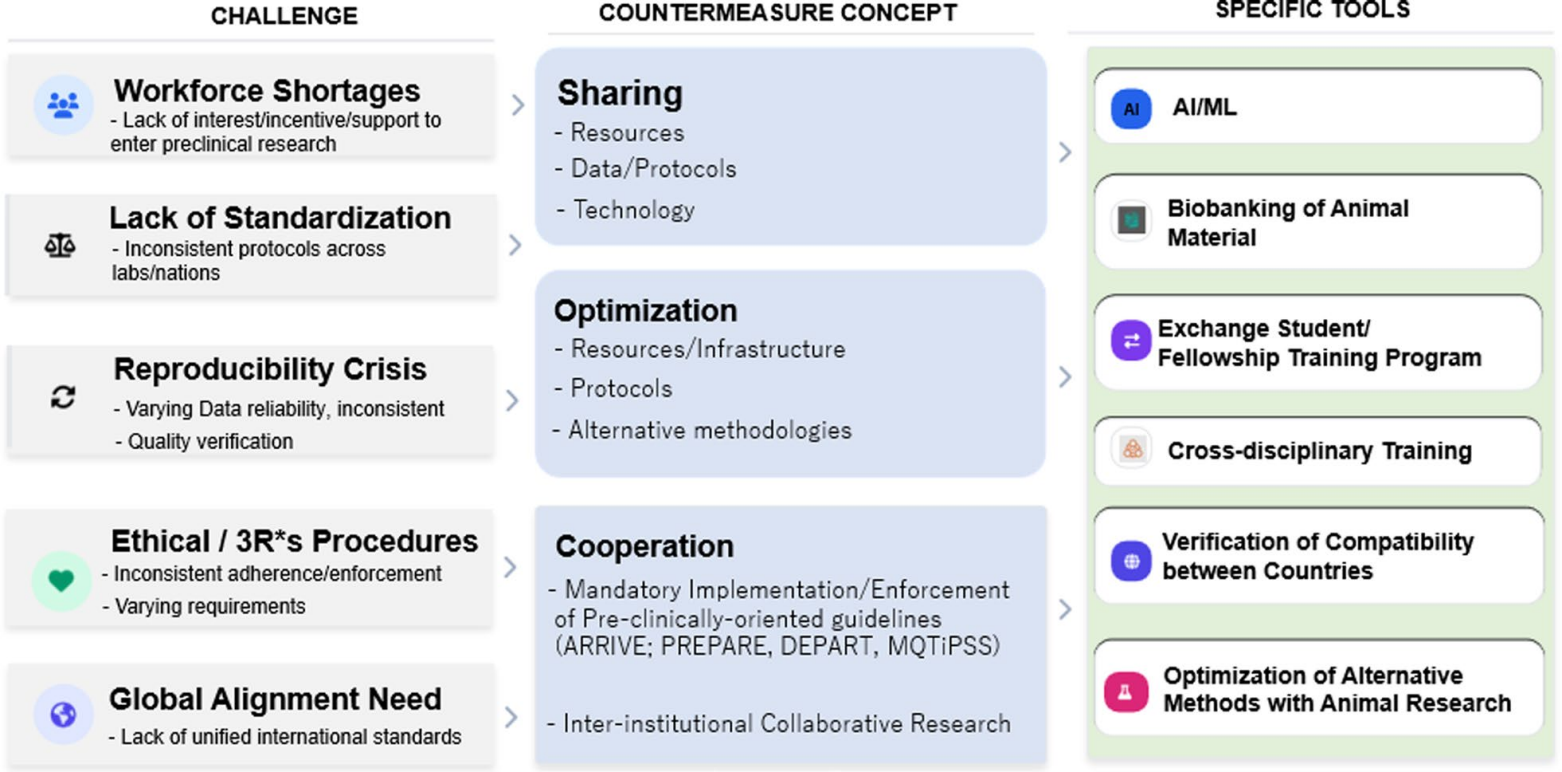
Practical guiding points to improve the field. Common challenges in the Asia–Pacific region were identified, and for each item, a countermeasure and specific tools that could serve as an actual solution are presented. *AI* artificial intelligence, *ML* machine learning, *ARRIVE* Animal Research: Reporting of In Vivo Experiments, *PREPARE* Planning Research and Experimental Procedures on Animals: Recommendations for Excellence, *DEPART* Detailed Experimental Protocols for Animal Research and Translation, *MQTiPSS* Minimum Quality Threshold in Preclinical Sepsis Studies, and *3R* Refinement, Reduction, Replacement

Although many challenges in animal model research could theoretically be addressed through international harmonization, establishing a single, globally unified regulatory framework is neither realistic nor necessarily desirable. The absence of full uniformity instead reflects legitimate regional differences in regulatory systems, resources, and research cultures. Nevertheless, international coordination remains essential to prevent unnecessary duplication of animal use and to avoid compromises in model selection or study design driven by local constraints or institutional isolation. To achieve this balance, key barriers—including the lack of internationally recognized oversight, auditing mechanisms, and shared quality benchmarks—must be addressed. In the Asia–Pacific region, several shared challenges have been identified. Workforce shortages, limited standardization, and reproducibility issues may be mitigated through the sharing and optimization of resources, data, protocols, and technologies. In contrast, challenges related to ethical oversight, implementation of the 3Rs, and global alignment require strengthened international collaboration (Fig. 3). We believe that countries and even regions should share their solvable and unsolvable challenges, progressing toward building collaborative networks rather than forcing them into uniform constraints.

Animal model research inherently involves biological variability, posing fundamental challenges to experimental consistency. It is self-evident that not all studies using animal models of critical illness have resulted in clinically translatable therapies [193]. Even highly refined trauma models allow direct extrapolation to human clinical trajectories [194]. These limitations, however, do not negate the fundamental value of animal research—an overwhelming number of technologies/solutions currently utilized in modern intensive care have been introduced and/or refined thanks to preclinical studies [195]. To ensure experimental consistency/reliability, it is important to address preventable sources of instability, such as procedural errors, inter-institutional variability, and over-reliance on single-center experimental designs, which collectively undermine reproducibility and generalizability. While standardization is essential for improving research quality, preserving space for innovative and creative model development remains a core mission of animal research, particularly for elucidating complex disease states.

Against this backdrop, coordinated and strategic efforts are required to enhance scientific rigor, reproducibility, and translational applicability. The National Centre for the Replacement, Refinement and Reduction of Animals in Research (NC3Rs) reported that only 59% of animal research articles adequately describe key study elements, such as objectives, animal numbers, and basic animal characteristics [196]. To address this, the ARRIVE (Animal Research: Reporting of In Vivo Experiments) guidelines were introduced in 2010 [197] and updated as ARRIVE 2.0 in 2020, incorporating the “Essential 10” and a complementary “Recommended Set” [197]. However, meta-analyses indicate that adherence to these guidelines remains suboptimal, raising persistent concerns regarding reproducibility [198]. Beyond general frameworks, such as ARRIVE, DEPART, and PREPARE, disease-specific preclinical guidelines have been developed for multiple conditions, including epilepsy, malaria, stroke, bone fractures, and sepsis [16, 199–202]. Ideally, these guidelines should be integrated from the earliest stages of experimental planning rather than treated as post hoc compliance requirements. In practice, however, implementation mechanisms such as protocol preregistration and enhanced peer review are time-consuming and rarely supported by dedicated funding, imposing additional burdens on researchers. Establishing consistent adherence across diverse geographical settings while keeping pace with scientific and ethical evolution will require sustained international coordination. In this context, international collaborative research represents a pragmatic pathway forward. Multicenter preclinical initiatives in traumatic brain injury have demonstrated the value of coordinated model development and validation [203]. A similar approach is warranted for critical illness models—including sepsis, ARDS, shock, and multiple-organ dysfunction—through the establishment of an Asia–Pacific preclinical research consortium to harmonize surgical techniques, outcome measures, and model validation.

Furthermore, to address persistent challenges, such as researcher overwork and the outflow of skilled personnel, robust international collaboration that equitably strengthens participating partners is essential. As a first step, a transnational network of researchers experienced in preclinical animal research should be established within the Asia–Pacific region and subsequently expanded to Europe and North America. Such collaborative structures would facilitate joint research initiatives, guideline standardization, sharing of advanced technologies and resources, and cross-validation of animal models in multinational settings.

The cultivation of early career researchers is central to sustaining rigor, reproducibility, and innovation in animal model research. Beyond technical expertise in laboratory animal science, researchers must possess a solid understanding of ethical principles and methodological foundations to select appropriate models based on their strengths, limitations, and translational relevance. For example, veterinarians are uniquely qualified to meet these requirements, and their integration into the core of research teams is highly desirable to strengthen human resources and improve scientific quality. While veterinarians in Europe and North America are often institutionally positioned to participate in scientific decision-making at the level of study leadership, their roles in the Asia–Pacific region remain largely confined solely to animal welfare and methodological support. This disparity reflects structural limitations in regional veterinary career pathways, particularly the lack of systems that foster veterinarians as independent investigators.

Emerging biomedical engineering approaches such as microfluidics, biosensors, advanced imaging, and organoid systems have the potential to create entirely new experimental paradigms [204]. However, these technologies are often costly, limited to specialized centers, and require deep interdisciplinary expertise. In contrast, AI and biobanking infrastructures offer more scalable opportunities to enhance rigor, reproducibility, animal welfare, and international collaboration. Biobanking cannot be implemented effectively at a global scale from the outset. Instead, regionally grounded biobanks supported by local funding agencies and legislative frameworks should first be established and subsequently interconnected into cooperative international networks.

To ensure the effective and responsible use of these approaches, governance frameworks are required to provide appropriate ethical oversight, data-sharing mechanisms, and accountability across institutions and borders. Collectively, these coordinated efforts can support a balanced and resilient research ecosystem that advances scientific quality while meeting evolving ethical expectations in animal model research.

## Conclusion

Animal models remain indispensable to critical care research, but their sustainability depends on improving reproducibility, ethical accountability, standardization, and workforce stability. In the Asia–Pacific region, functional international alignment—rather than rigid global uniformity—can balance regional diversity with transparency and comparability. Integrating globally recognized frameworks with emerging approaches will be essential to enhance translational relevance, animal welfare, and long-term research capacity.

## Data Availability

Not applicable. This study is a narrative review and does not involve the generation or analysis of new data sets.
